# Regional Disparities in Measles Vaccination Coverage and Their Associated Factors: An Ecological Study in Japan

**DOI:** 10.2188/jea.JE20240129

**Published:** 2025-02-05

**Authors:** Masaki Machida, Shinji Fukushima, Takahiro Tabuchi, Tomoki Nakaya, Wakaba Fukushima, Shigeru Inoue

**Affiliations:** 1 Department of Preventive Medicine and Public Health, Tokyo Medical University, Tokyo, Japan; 2 Department of Infection Prevention and Control, Tokyo Medical University Hospital, Tokyo, Japan; 3 Travellers’ Medical Center, Tokyo Medical University Hospital, Tokyo, Japan; 4 Division of Epidemiology, School of Public Health, Tohoku University Graduate School of Medicine, Sendai, Japan; 5 Graduate School of Environmental Studies, Tohoku University, Sendai, Japan; 6 Graduate School of Science, Tohoku University, Sendai, Japan; 7 Department of Public Health, Osaka Metropolitan University Graduate School of Medicine, Osaka, Japan

**Keywords:** measles vaccination, single-parent household, area deprivation index, socioeconomic status, Japan

## Abstract

**Background:**

The decline in measles vaccination coverage is a global concern. In Japan, coverage of the first dose of measles vaccine, which had exceeded the target of 95.0% since fiscal year (FY) 2010, fell to 93.5% in FY 2021. Vaccination coverage increased to 95.4% in FY 2022 but varied by municipality. Few studies have focused on regional disparities in measles vaccination coverage. This study aimed to clarify the regional disparities in measles vaccination coverage by municipality in Japan and their associated factors.

**Methods:**

In this ecological study, the measles vaccination coverage in FY 2022; population density; area deprivation index (ADI; an indicator of socioeconomic status); proportion of foreign nationals, single-father households, single-mother households, and mothers aged ≥30 years; and number of medical facilities, pediatricians, and non-pediatric medical doctors in 1,698 municipalities were extracted from Japanese government statistics. Negative binomial regression was performed with the number of children vaccinated against measles as the dependent variable, number of children eligible for measles vaccination as the offset term, and other factors as independent variables.

**Results:**

Vaccination coverage was less than 95.0% in 54.3% of municipalities. Vaccination coverage was significantly positively associated with population density and negatively associated with the proportion of single-father households, mothers aged ≥30 years, and the ADI (incidence rate ratios: 1.004, 0.976, 0.999, and 0.970, respectively).

**Conclusion:**

This study showed regional disparities in measles vaccination coverage in Japan. Single-father households, age of mothers, and socioeconomic status may be key factors when municipalities consider strategies to improve vaccination coverage.

## INTRODUCTION

Measles is a highly contagious, potentially fatal, and vaccine-preventable disease caused by the measles virus.^1^ Measles vaccination is included in the National Immunization Program (NIP) as routine immunizations of many countries. Measles elimination, defined as the absence of endemic measles transmission, was achieved in Japan in 2015, although imported measles cases have caused outbreaks in several regions.^2^ The number of measles cases in Japan was low from 2020 to 2022, when the coronavirus disease 2019 (COVID-19) pandemic reduced international migration, but has increased since 2023 as migration began to return to normal.^3^ Measles vaccination coverage of at least 95.0% is required to maintain herd immunity and prevent epidemics.^4^ However, recently, declining measles vaccination coverage has become a global concern. The World Health Organization (WHO) reported a sustained decline in the coverage of the first dose of measles vaccine, falling to 81% in 2021, which is the lowest level since 2008.^5^ In Japan as a whole, the coverage of the first dose of measles vaccine, which had been above 95.0% since fiscal year (FY) 2010, fell to 93.5% in FY 2021.^6^ In FY 2022, the coverage improved to 95.4%, but varied by municipality. Regional disparities in vaccination coverage contribute to health inequalities by affecting the morbidity, severity, and mortality rates of vaccine-preventable diseases. However, few studies have focused on regional disparities in vaccination coverage.^7^^–^^9^ A previous study in the United States reported socioeconomic status (SES), ethnicity, and proportion of single-parent households as factors associated with regional disparities in COVID-19 vaccination coverage among adults.^7^ Some social demographic factors, such as age, sex, ethnicity, and SES, are associated with individuals’ vaccination willingness and motivation to be vaccinated.^10^ In addition to individual vaccination intentions and motivation, the accessibility of immunization services, such as ease of access and availability, also influences individual vaccination behavior.^11^^,^^12^ Previous studies on regional disparities in vaccination coverage have not assessed the accessibility of vaccination services. Additionally, to the best of our knowledge, no studies have assessed the factors contributing to regional disparities in measles vaccination coverage.

Therefore, we performed an ecological study using official statistics of Japan to clarify regional disparities in measles vaccination coverage in Japan and their associated factors.

## METHODS

### Data source

In Japan, measles vaccination is included in the NIP as routine immunizations and is administered at the municipal level. Therefore, this exploratory study used an ecological study design to clarify the factors associated with municipality-level measles vaccination coverage. We assessed associated sociodemographic factors identified in previous studies and potential factors related to the accessibility of immunization services as independent variables.^7^^–^^10^ Vaccination coverage was obtained from publicly available information from the Ministry of Health, Labour and Welfare,^6^ and data on the independent variables for each municipality were obtained from e-State, the portal site for Japanese government statistics.^13^ Because only open-access aggregated data were used, ethical approval was not required.

### Dependent variable

We used the first dose measles vaccination coverage among 1-year-old children in Japan in FY 2022 by municipality. FY 2022 was chosen because regional disparities in vaccination coverage became pronounced in FY 2022. The measles vaccination coverage was calculated by dividing the number of children aged 1 year who received the measles vaccine in FY 2022 by the number of children eligible for the first dose of measles vaccine as of October 1, 2022.^14^ As coverage calculated by this method can exceed 100%, in this study, coverage exceeding 100% was converted to 100% in basic statistics.

### Independent variables

Population (number per municipality), population density (defined as the number of people per square kilometer of land area; number/km^2^), proportion of foreign nationals (%), number of single-father households, number of single-mother households, and number of households with children, were extracted from the results of the 2020 census, which was the most recent figure available. Population density was subjected to a logarithmic transformation using the natural logarithm due to its right-skewed distribution. Each one-unit increase in the logarithm of population density corresponds to a population density (number/km^2^) increase of approximately 2.72 times. The number of single-father and single-mother households divided by the number of households with children was defined as the proportion of single-father and single-mother households (%), respectively. The area deprivation index (ADI), derived from the 2020 census results, was used as an indicator of SES at the municipal level. This composite indicator comprises the weighted sums of poverty-related census variables. A higher ADI score indicates greater deprivation in the municipality. Details of the calculation of ADI are described elsewhere.^15^ Information on the age of mothers of children aged 1 year eligible for vaccination, was calculated from the mother’s age at the birth of their children reported in the FY 2021 vital statistics. The proportion of mothers aged ≥30 years (%) was calculated based on a previous study’s findings that vaccine refusal is more common among mothers in their 30s and 40s.^16^

As potential factors related to the accessibility of immunization services, the number of medical facilities was extracted from a survey of medical institutions conducted in FY 2022, and the number of pediatricians and non-pediatric medical doctors was extracted from the statistics of physicians, dentists and pharmacists conducted in FY 2020, which were the most recent data available. The number of medical facilities per habitable land area (number per 1 km^2^) was calculated by dividing the number of medical facilities by the habitable land area (km^2^), which was extracted from statistical observations of municipalities 2022. The number of pediatricians per 1,000 population and the number of non-pediatric medical doctors per 1,000 population (number per 1,000 population) were calculated using the census results.

### Statistical analysis

We excluded municipalities with 1) no children eligible for measles vaccination, 2) census and measles coverage using different units of disaggregation, and 3) ADI that could not be calculated. Each indicator was compared between two groups of municipalities—those with measles vaccination coverage of 95% or higher and those with less than 95%*—*using *t*-tests. Negative binomial regression was performed to clarify factors associated with measles vaccination coverage. The dependent variable was the number of children aged 1 year who received the measles vaccine in FY 2022. The independent variables were population density, ADI, the proportion of foreign nationals, single-father households, single-mother households, and mothers aged ≥30 years; and the number of medical facilities, pediatricians, and non-pediatric medical doctors. The offset term was the number of children eligible for the first dose of measles vaccine as of October 1, 2022. The incidence rate ratio (IRR) was calculated. Additionally, to clarify trends according to mothers’ age, negative binomial regression was conducted with the mothers’ age divided into two proportions with different cut-off points: proportion of mothers aged 30 to 39 years, and proportion of mothers aged ≥40 years. For sensitivity analysis, considering unknown regional tendencies of vaccination, an additional multivariable negative binomial regression was performed as a sensitivity analysis by adding typical regional divisions (ie, Hokkaido, Tohoku, Kanto, Chubu, Kinki, Chugoku, Shikoku, and Kyushu) as a set of eight dummy variables.^17^ R version 4.2.0 (using “MASS” from the R package; R Foundation for Statistical Computing, Vienna, Austria) was used to perform the statistical analyses, with statistical significance defined as two-sided *P*-values <0.05.

## RESULTS

The number of municipalities with published measles vaccination coverage was 1,737. Of these, 39 were excluded from the analysis. We excluded 4 municipalities with no children eligible for measles vaccination, 22 municipalities with census and vaccination coverage using different units of disaggregation, and 13 municipalities for which it was not possible to calculate the ADI; therefore, the final dataset used for the analysis included 1,698 municipalities.

**Figure 1.**
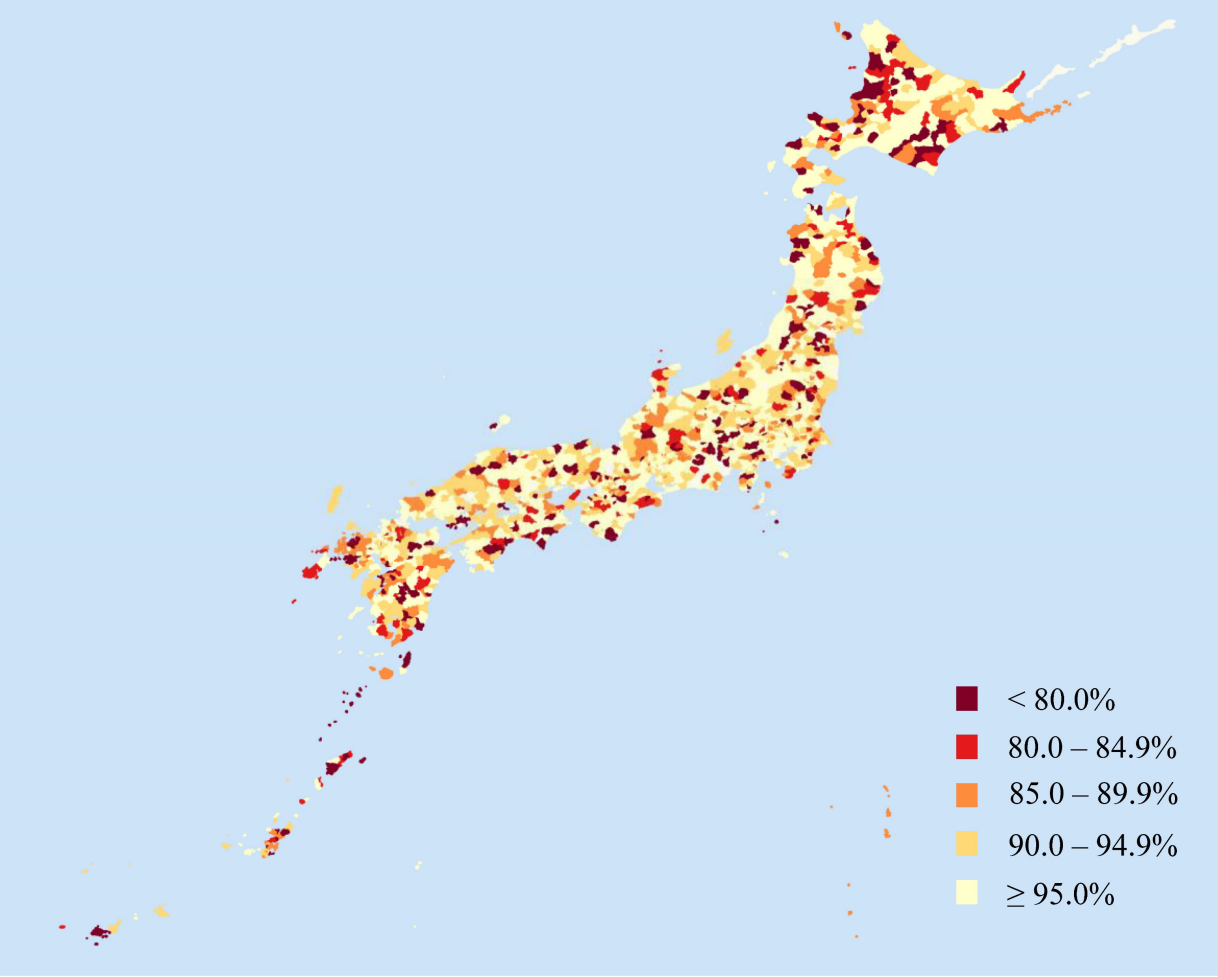
Measles vaccine coverage by municipality in Japan

The basic statistics for each indicator by municipality are shown in Table 1. The mean measles vaccination coverage by municipality was 91.2% (range: 0–100%, 50th percentile: 94.2%); however, values varied widely by municipality (Figure 1). Among the municipalities, 54.3% (*n* = 922) had less than 95.0% measles vaccination coverage. Municipalities with less than 95% measles vaccination coverage had lower population density, lower proportion of foreign nationals, fewer medical facilities, fewer pediatricians, and higher ADI than municipalities with more than 95% measles vaccination coverage (Table 2).

**Table 1.** Basic statistics of each indicator by municipality

Indicators (units)	Mean	SD	Min	Max	25th percentile	50th percentile	75th percentile
Population (number)	57,840.45	96,808.36	323.00	943,664.00	7,712.50	23,393.00	60,861.00
Number of measles vaccine-eligible persons (number)	376.44	673.58	1.00	6,299.00	36.00	123.00	387.50
Measles vaccination coverage (%)	91.20	10.90	0.00	100.00	87.90	94.20	99.10
Population density^a^	5.29	1.88	0.47	10.05	3.97	5.24	6.61
Proportion of foreign nationals (%)	1.44	1.47	0.00	18.99	0.61	1.01	1.76
Proportion of single-father households (%)	0.59	0.40	0.00	4.35	0.37	0.50	0.69
Proportion of single-mother households (%)	4.41	2.16	0.00	23.54	3.01	3.96	5.28
Area deprivation index^b^	6.13	0.66	3.64	9.56	5.71	6.07	6.55
Number of mothers aged ≥30 years (number)	239.46	461.82	0.00	5,476.00	22.00	73.00	233.00
Proportion of mothers aged ≥30 years (%)	63.57	10.29	0.00	100.00	58.64	63.16	67.88
Number of medical facilities per square kilometer of habitable land area (number per 1 km^2^)	1.26	3.74	0.00	72.18	0.19	0.39	0.96
Number of pediatricians (number per 1,000 population)	0.07	0.10	0.00	1.07	0.00	0.05	0.09
Number of non-pediatric medical doctors (number per 1,000 population)	1.71	1.89	0.00	27.29	0.79	1.36	2.04

SD, standard deviation.

^a^Population density, defined as the number of people per square kilometer of land area (number/km^2^), was subjected a logarithmic transformation using the natural logarithm due to its right-skewed distribution. This indicator is unitless. Each one-unit increase in the logarithm of population density corresponds to a population density (number/km^2^) increase of approximately 2.72 times.

^b^The area deprivation index, derived from the 2020 census results, was used as an indicator of socioeconomic status at the municipal level. This composite indicator comprises the weighted sums of poverty-related census variables. This indicator is unitless.

**Table 2.** Comparison of indicators between municipalities with measles vaccination coverage of 95% or higher and those with less than 95%

Indicators (units)	Measles vaccination coverage: 95% or higher	Measles vaccination coverage: less than 95%	*P*-value^a^
	(*n* = 776)	(*n* = 922)	
	Mean (SD)	Mean (SD)	
Population density^b^	5.42 (2.03)	5.19 (1.73)	0.012
Proportion of foreign nationals (%)	1.55 (1.50)	1.35 (1.43)	0.005
Proportion of single-father households (%)	0.58 (0.43)	0.59 (0.38)	0.461
Proportion of single-mother households (%)	4.31 (2.06)	4.49 (2.24)	0.096
Area deprivation index^c^	6.05 (0.69)	6.19 (0.63)	<0.001
Proportion of mothers aged ≥30 years (%)	63.80 (11.00)	63.39 (9.64)	0.416
Number of medical facilities per square kilometer of habitable land area (facilities per 1 km^2^)	1.71 (5.03)	0.88 (2.04)	<0.001
Number of pediatricians (number per 1,000 population)	0.07 (0.11)	0.06 (0.09)	0.042
Number of non-pediatric medical doctors (number per 1,000 population)	1.78 (2.16)	1.65 (1.62)	0.145

SD, standard deviation.

^a^*P*-value was calculated using *t*-tests.

^b^Population density, defined as the number of people per square kilometer of land area (number/km^2^), was subjected a logarithmic transformation using the natural logarithm due to its right-skewed distribution. This indicator is unitless. Each one-unit increase in the logarithm of population density corresponds to a population density (number/km^2^) increase of approximately 2.72 times.

^c^The area deprivation index, derived from the 2020 census results, was used as an indicator of socioeconomic status at the municipal level. This composite indicator comprises the weighted sums of poverty-related census variables. This indicator is unitless.

Table 3 shows the results of negative binomial regression. The population density (IRR 1.004; 95% confidence interval [CI], 1.001–1.006), and the proportion of foreign nationals (IRR 1.002; 95% CI, 1.000–1.005) demonstrated a significant positive association with measles vaccination coverage. The proportion of single-father households (IRR 0.976; 95% CI, 0.954–0.999), ADI (IRR 0.970; 95% CI, 0.960–0.980), and the proportion of mothers aged ≥30 years (IRR 0.999; 95% CI, 0.998–0.999) demonstrated a significant negative association with measles vaccination coverage. In the analysis with mothers’ age divided into two proportions with different cut-off points, both the proportion of mothers aged 30–39 years (IRR 0.999; 95% CI, 0.998–1.000) and the proportion of mothers aged ≥40 years (IRR 0.997; 95% CI, 0.995–0.999) demonstrated a significant negative association with measles vaccine coverage (eTable 1). The multivariable model including eight regions as a sensitivity analysis showed similar significant associations between measles vaccination coverage and population density, proportion of single-father households, ADI, and proportion of mothers aged ≥30 years (eTable 2).

**Table 3.** Factors associated with measles vaccination coverage

Indicators (units)	Negative binomial regression model^a^			

	Univariable model		Multivariable model^b^	

	IRR (95% CI)	*P*-value	IRR (95% CI)	*P*-value
Population density^c^	1.009 (1.007–1.011)	<0.001	1.004 (1.001–1.006)	0.011
Proportion of foreign nationals (%)	1.006 (1.004–1.008)	<0.001	1.002 (1.000–1.005)	0.033
Proportion of single-father households (%)	0.946 (0.931–0.961)	<0.001	0.976 (0.954–0.999)	0.042
Proportion of single-mother households (%)	0.994 (0.992–0.996)	<0.001	1.002 (0.999–1.006)	0.228
Area deprivation index^d^	0.972 (0.967–0.977)	<0.001	0.970 (0.960–0.980)	<0.001
Proportion of mothers aged ≥30 years (%)	1.001 (1.000–1.001)	<0.001	0.999 (0.998–0.999)	<0.001
Number of medical facilities per habitable land area (number per 1 km^2^)	1.002 (1.001–1.002)	<0.001	1.000 (0.999–1.001)	0.954
Number of pediatricians (number per 1,000 population)	1.061 (1.028–1.094)	<0.001	1.003 (0.955–1.053)	0.911
Number of non-pediatric medical doctors (number per 1,000 population)	1.003 (1.001–1.004)	<0.001	1.000 (0.998–1.003)	0.850

CI, confidence interval; IRR, incidence rate ratio.

^a^The dependent variable was the number of children aged 1 year who received the measles vaccine in 2022. The offset term was the number of children eligible for the first dose of measles vaccine as of October 1, 2022.

^b^The independent variables were all variables in the Table.

^c^Population density, defined as the number of people per square kilometer of land area (number/km^2^), was subjected a logarithmic transformation using the natural logarithm due to its right-skewed distribution. This indicator is unitless. Each one-unit increase in the logarithm of population density corresponds to a population density (number/km^2^) increase of approximately 2.72 times.

^d^The area deprivation index, derived from the 2020 census results, was used as an indicator of socioeconomic status at the municipal level. This composite indicator comprises the weighted sums of poverty-related census variables. This indicator is unitless.

## DISCUSSION

Our results showed that measles vaccination coverage was less than 95% in more than half of the municipalities and low in municipalities with a low population density, high population of single-father households, mothers aged ≥30 years, and worse municipality deprivation. The IRR for the proportion of mothers aged ≥30 years was 0.999, indicating that a 10% increase in this proportion is associated with approximately a 1.00% relative decrease in measles vaccination coverage ([1 − 0.999^10^] × 100). For instance, if the current vaccination coverage is 95%, it would be reduced to approximately 94.05% (95% × 0.99). The number of medical institutions and doctors were not significantly associated with measles vaccination coverage in the negative binomial regression.

Vaccine hesitancy, defined as the delay in acceptance or refusal of vaccination despite the availability of vaccination services, is a complex issue influenced by various factors.^10^ The WHO has proposed the Behavioural and Social Drivers of vaccination (BeSD) framework to understand the factors influencing vaccine uptake.^11^^,^^12^ In this framework, individuals’ “thinking and feeling” regarding vaccines (eg, vaccine confidence and perceived disease risk) and “social processes” (eg, social norms and health worker recommendations) form “motivation” towards vaccines, and “motivation” and “practical issues” (eg, ease of access) determine whether to receive a vaccine.^11^^,^^12^ In the BeSD framework, individuals with vaccine hesitancy are classified into two main types: those with low motivation regarding vaccines, and those with a high motivation to be vaccinated but delay or do not receive the vaccination due to inaccessibility of vaccination services. Previous studies have reported lower vaccination motivation among young people, women, and those with low SES.^10^^,^^18^ Previous studies on regional disparities in vaccination coverage have also reported SES as factor contributing to regional disparities.^7^^–^^9^ In this study, ADI, which was used as an indicator of SES, was associated with disparities in vaccination coverage. A recent internet survey in Japan indicated that adults in their 30s and 40s were more likely than those in other age groups to refuse general vaccination.^16^ In this study using government statistics, a consistent negative trend in vaccination coverage was observed with increasing proportions of mothers in older age groups. The low measles vaccination coverage in municipalities with a high proportion of mothers aged ≥30 years may also reflect low vaccination motivation within the community as a whole.

Men are more likely to have positive attitudes towards general vaccination than women.^10^^,^^16^^,^^18^ Meanwhile, in this study, measles vaccination coverage was low in municipalities with a high proportion of single-father households. A previous study conducted in the United States reported lower COVID-19 vaccination coverage in regions with a higher proportion of single-parent households, and the authors attributed inaccessibility of vaccination services for single parents as a factor contributing to the lower coverage.^7^ However, the previous study did not examine differences according to the parental sex.^7^ In this study, the proportion of single-mother households was not associated with measles vaccination coverage in the main analysis, although the sensitivity analysis showed a significant positive association between the proportion of single-mother households and measles vaccination coverage. Conversely, the proportion of single-father households was negatively associated with the measles vaccination coverage both in the main analysis and the sensitivity analysis. These results suggest that the influence of single-parent households on vaccination coverage varies by parental sex, with single-father households potentially having a more negative impact on vaccination coverage.

Urban areas tend to have higher vaccination coverage than rural areas.^7^^,^^19^ Previous studies have reported that vaccination coverage may be lower in rural areas than in urban areas due to greater barriers to accessing health services than in urban areas.^7^^,^^20^ In this study, population density was also positively associated with vaccination coverage, consistent with previous studies. In the sensitivity analysis that included region, the proportion of foreign nationals was not associated with the coverage. This may also be related to the degree of urbanization, as the proportion of foreign nationals tends to be higher in regions that include urban areas. However, this study showed no significant association between measles vaccination coverage and the number of health facilities and medical doctors in the municipality. In this study, the number of medical institutions and doctors divided by the habitable land area and population were used as surrogate of accessibility of vaccination services. However, the accessibility of vaccination services may need to be assessed by more detailed indicators, such as the distance from the place of residence and doctors’ office hours. Further research is needed to examine the effect of the accessibility of vaccination services on vaccination coverage, based on a more detailed analysis of the location of health facilities.

To the best of our knowledge, this is the first study to focus on regional disparities in measles vaccination coverage and to clarify that vaccination coverage is lower in areas with a higher proportion of single-father households, and in areas with a higher proportion of mothers aged ≥30 years. However, this study has several limitations. First, the method used to calculate measles vaccination coverage has limitations. In this study, vaccination coverage was calculated by dividing the number of children aged 1 year who received the measles vaccine in 2022 by the number of children eligible for the first dose of the measles vaccine as of October 1, 2022.^14^ This calculation method did not consider eligible children who had been vaccinated in a different municipality due to relocation or other reasons. Second, the results of the census and the statistics of physicians, dentists and pharmacists were based on FY 2020 data, which is different from the measles vaccination coverage data. The census was not conducted in FY 2022, and the results of the statistics of physicians, dentists and pharmacists for FY 2022 were not available at the time of the study. Third, this study did not consider whether single-father and single-mother households had a child eligible for the first dose of measles vaccine. The census of Japan defines single-father and single-mother households as nuclear family households consisting only of unmarried, widowed, or separated female or male parents and their unmarried children aged <20 years.^21^ Further research is needed to clarify the association between household composition and vaccination behavior. Fourth, the proportion of mothers aged ≥30 years was calculated based on mother’s age at birth of children in the previous year. This could differ from the actual proportion of mothers aged ≥30 years with 1-year-old children eligible for the first dose of measles vaccine in FY 2022. Fifth, FY2022 falls within the timeframe of the COVID-19 pandemic, which may have impacted the results of this study. Given the variability of vaccine coverage and vaccine hesitancy over time,^10^ continual research is needed to timely determine the regional disparities. Sixth, it remains unclear whether the findings of this study are specific to the measles vaccination or applicable to other childhood vaccinations. Notably, Japan had a vaccine gap until 2013, which denotes a delay in the Japanese immunization program compared to programs in other developed countries.^22^^,^^23^ Although the vaccine gap has shown improvements in recent years, mumps vaccine continues to have a vaccine gap.^23^ The mumps vaccine remines categorized as voluntary vaccination in Japan and the vaccination coverage is unclear. Regarding human papillomavirus (HPV) vaccine, it was included in the NIP in April 2013. However, the Ministry of Health, Labour and Welfare suspended the proactive recommendation of the HPV vaccination in June 2013 because concerns about vaccine-related adverse events were widely reported in the Japanese mass media.^24^^,^^25^ In April 2022, the Ministry of Health, Labour and Welfare has resumed the recommendation for proactive HPV vaccination and simultaneously initiated catch-up vaccinations^26^^,^^27^; however, vaccine coverage remains low.^28^ Future research on vaccination coverage and regional disparities related to these vaccines is also warranted. Seventh, this was an ecological study and is susceptible to the ecological fallacy. Despite these limitations, this study provides useful information that could be used to reduce regional disparities in measles vaccination coverage in Japan.

In conclusion, this study showed regional disparities in measles vaccination coverage in Japan. Single-father households, age of mothers, and socioeconomic status may be key factors when municipalities consider strategies to improve vaccination coverage.

## SUPPLEMENTARY MATERIAL

eTable 1. Results of the negative binomial regression model with the age of mothers divided into two proportions with different cut-off points (30–39 years, ≥40 years)

eTable 2. Results of the negative binomial regression model including eight regions in Japan as dummy variables
