# Supplementary material for: Regional Disparities in Measles Vaccination Coverage and Their Associated Factors: An Ecological Study in Japan
Source: J Epidemiol. 2025 Feb 5;35(2):100–5. doi: 10.2188/jea.JE20240129 (PMC11706677; doi:10.2188/jea.JE20240129)
Supplement: Supplementary file 1 [file je-35-100-s001.pdf]

eTable 1. Results of the negative binomial regression model with the age of mothers divided into two proportions with different cut-off points (30–39 years, ≥40 years)

	Multivariable negative binomial regression model [a]	
Indicators (units)	IRR (95% CI)	*P*-value
Population density [b]	1.004 (1.001–1.006)	0.010
Proportion of foreign nationals (%)	1.002 (1.000–1.005)	0.041
Proportion of single-father households (%)	0.978 (0.955–1.001)	0.061
Proportion of single-mother households (%)	1.002 (0.999–1.006)	0.229
Area deprivation index [c]	0.971 (0.961–0.981)	<0.001
Proportion of mothers aged 30 to 39 years (%)	0.999 (0.998–1.000)	0.005
Proportion of mothers aged ≥40 years (%)	0.997 (0.995–0.999)	<0.001
Number of medical facilities per habitable land area (number per 1 km^2)	1.000 (0.999–1.001)	0.613
Number of pediatricians (number per 1,000 population)	1.003 (0.956–1.054)	0.889
Number of non-pediatric medical doctors (number per 1,000 population)	1.000 (0.998–1.003)	0.905

CI, confidence interval; IRR, incidence rate ratio.

[a] The dependent variable was the number of children aged 1 year who received the measles vaccine in 2022. The independent variables consisted of all variables listed in the Table. The offset term was the number of children eligible for the first dose of measles vaccine as of 1st October 2022.

[b] Population density, defined as the number of people per square kilometer of land area (number/km^2), was subjected a logarithmic transformation using the natural logarithm due to its right-skewed distribution. This indicator is unitless. Each one-unit increase in the logarithm of population density corresponds to a population density (number/km^2) increase of approximately 2.72 times.

[c] The area deprivation index, derived from the 2020 census results, was used as an indicator of socioeconomic status at the municipal level. This composite indicator comprises the weighted sums of poverty-related census variables. This indicator is unitless.

eTable 2. Results of the negative binomial regression model including eight regions in Japan as dummy variables

Indicators (units)	Multivariable negative binomial regression model [a]	
	IRR (95% CI)	*P*-value
Population density [b]	1.006 (1.003−1.009)	<0.001
Proportion of foreign nationals (%)	1.002 (0.999−1.004)	0.191
Proportion of single-father households (%)	0.975 (0.952−0.999)	0.040
Proportion of single-mother households (%)	1.004 (1.000−1.008)	0.041
Area deprivation index [c]	0.973 (0.963−0.983)	<0.001
Proportion of mothers aged ≥30 years (%)	0.999 (0.998−0.999)	<0.001
Number of medical facilities per habitable land area (number per 1 km^2)	1.000 (0.999−1.001)	0.869
Number of pediatricians (number per 1,000 population)	1.002 (0.955−1.052)	0.932
Number of non-pediatric medical doctors (number per 1,000 population)	1.000 (0.998−1.003)	0.778
Region [d]		
Hokkaido	Ref	
Tohoku	0.996 (0.975−1.018)	0.746
Kanto	0.987 (0.966−1.009)	0.245
Chubu	0.995 (0.974−1.016)	0.636
Kinki	0.980 (0.960−1.001)	0.062
Chugoku	0.996 (0.974−1.019)	0.744
Shikoku	0.995 (0.971−1.019)	0.672
Kyushu	0.967 (0.949−0.986)	<0.001

CI, confidence interval; IRR, incidence rate ratio.

[a] The dependent variable was the number of children aged 1 year who received the measles vaccine in 2022. The independent variables consisted of all variables listed in the Table. The offset term was the number of children eligible for the first dose of measles vaccine as of 1st October 2022.

[b] Population density, defined as the number of people per square kilometer of land area (number/km^2), was subjected a logarithmic transformation using the natural logarithm due to its right-skewed distribution. This indicator is unitless. Each one-unit increase in the logarithm of population density corresponds to a population density (number/km^2) increase of approximately 2.72 times.

[c] The area deprivation index, derived from the 2020 census results, was used as an indicator of socioeconomic status at the municipal level. This composite indicator comprises the weighted sums of poverty-related census variables. This indicator is unitless.

[d] The prefectures in each region are as follows: Hokkaido: Hokkaido; Tohoku: Aomori, Iwate, Akita, Miyagi, Yamagata, Fukushima; Kanto: Ibaraki, Tochigi, Gunma, Saitama, Chiba, Tokyo, Kanagawa; Chubu: Niigata, Ishikawa, Toyama, Fukui, Yamanashi, Nagano, Gifu, Shizuoka, Aichi; Kinki: Mie, Shiga, Kyoto, Osaka, Hyogo, Nara, Wakayama; Chugoku: Tottori, Shimane, Okayama, Hiroshima, Yamaguchi; Shikoku: Tokushima, Kagawa, Ehime, Kochi; Kyushu: Fukuoka, Saga, Nagasaki, Kumamoto, Oita, Miyazaki, Kagoshima, Okinawa
